# Inoculation with *Azospirillum brasilense* and/or *Pseudomonas geniculata* reinforces flax (*Linum usitatissimum*) growth by improving physiological activities under saline soil conditions

**DOI:** 10.1186/s40529-022-00345-w

**Published:** 2022-05-19

**Authors:** Amal M. Omer, Mahmoud S. Osman, Ali A. Badawy

**Affiliations:** 1 Soil Fertility and Microbiology Department, Desert Research Center, El-Matareya 11753, Cairo, Egypt; 2 Botany and Microbiology Department, Faculty of Science, Al-Azhar University, Nasr City, Cairo, 11884 Egypt

**Keywords:** Endophytes, Flax plants, Salinized soil, Oxidative stress, Osmolytes, Antioxidant enzymes

## Abstract

**Background:**

Salinized soils negatively affect plant growth, so it has become necessary to use safe and eco-friendly methods to mitigate this stress. In a completely randomized design, a pot experiment was carried out to estimate the influence of the inoculation with endophytic bacterial isolates *Azospirillum brasilense*, *Pseudomonas geniculata* and their co-inoculation on growth and metabolic aspects of flax (*Linum usitatissimum*) plants that already grown in salinized soil.

**Results:**

The results observed that inoculation of salinity-stressed flax plants with the endophytes *A. brasilense* and *P. geniculata* (individually or in co-inoculation) increases almost growth characteristics (shoot and root lengths, fresh and dry weights as well as number of leaves). Moreover, contents of chlorophylls and carotenoids pigments, soluble sugars, proteins, free proline, total phenols, ascorbic acid, and potassium (K^+^) in flax plants grown in salinized soil were augmented because of the inoculation with *A. brasilense* and *P. geniculata*. Oppositely, there are significant decreases in free proline, malondialdehyde (MDA), hydrogen peroxide (H_2_O_2_), and sodium (Na^+^) contents. Regarding antioxidant enzymes, including superoxide dismutase (SOD), peroxidase (POD), and ascorbate peroxidase (APX), the inoculation with the tested endophytes led to significant enhancements in the activities of antioxidant enzymes in stressed flax plants.

**Conclusions:**

The results of this work showed that the use of the endophytic bacterial isolates *Azospirillum brasilense*, *Pseudomonas geniculata* (individually or in co-inoculation) could be regarded as an uncommon new model to alleviate salinity stress, especially in salinized soils.

## Introduction

The life of plants is constantly exposed to inappropriate conditions and various environmental stresses such as salinity, drought, chilling, heat, heavy metals, gaseous pollutants, pathogens, and other conditions that cause deleterious effects on the growth, metabolism, and productivity of various crops (Ewais et al. 2015; Zhang et al. 2018; Attia et al. 2021b; Hussein et al. 2022). Salinity is among the main problems which can reduce crop production in addition to its effect on germination rate, plant growth, and grain quality. Global indicators document that salt stress influences 22% of the overall agricultural output and 33% of the total irrigated land, forecasting a rise in salt stress to 10% per year (Ilyas et al. 2020). Resulting in a higher presence of sodium chloride (NaCl), plants growing in salt-affected soils have both hyper-osmotic and hyper-ionic effects. These stresses inhibit water absorption, reduce the absorption rate of ions and minerals, produce reactive oxygen species, which lead to a disturbance in the functions of the plasma membrane and a reduction in metabolic activities (Mishra et al. 2018; Abdel Latef et al. 2020) thus affecting growth features, morphology, pigments, biochemical constituents and survival of the plant (Zhang et al. 2014; Osman et al. 2021).

It is a worthy note that plants in the natural ecosystem live symbiotically with endophytic microorganisms, and these endophytes have a great role in enhancing the tolerance of the host plants to several stress conditions (Rodriguez et al. 2008; Redman et al. 2011). Endophytic bacteria are a set of heterogeneous microorganisms that occupy the internal of plant cells and therefore are considered to have positive impacts on growth and nutrition of the host plant (Schulz and Boyle 2006) without doing effective harm or gaining any benefit other than residency, and also without showing any external sign of infection on their host (Ahemad and Kibret 2014). Endophytic bacteria facilitate the growth and development of the plant through a variety of mechanisms similar to the plant growth-promoting rhizobacteria, including indole acetic acid (plant growth regulator) and siderophores production, nitrogen fixation, phosphate solubilization (Ryan et al. 2008; Glick 2012). Endophytes are closely interacting more than phyllosphere and rhizosphere bacteria with their host plants. To be in direct relationship with the plant, endophytic bacteria can directly or indirectly promote plant growth, particularly under various stress conditions (Weyens et al. 2009).

Azospirillum bacteria, which are gram-negative, are in the Spirillaceae family and are unable to produce internal spores (Fendrihan et al. 2017; Abdel Latef et al. 2020). Potential benefits of Azospirillum are primarily attributed to biochemical and anatomical improvements throughout the host plant roots contributing to the enhancement of water and mineral absorption (Bashan and De-Bashan 2010). Azospirillum affects the rate, and length of the hairy root, increasing the development of the lateral roots that enhance the root area (Fukami et al. 2018). The genus *Pseudomonas* belongs to the family Pseudomonadaceae, which is a Gram-negative bacterium that has been documented for its ability to form colonies with plant roots; thus it is a potential agent in the development of biological inoculants (Dekkers et al. 2000). Recent research has listed the position of *Pseudomonas* sp. in promoting plant growth and controlling nutrient accumulation and stress responses in the rhizosphere (Samaddar et al. 2019). Azospirillum and Pseudomonas are two of the most frequently microbial genera that have shown the ability to colonize the plant rhizosphere and enhance plant growth (Saharan and Nehra 2011) also produce indole acetic acid (IAA) under salt stress (Egamberdieva et al. 2008, 2019). Azospirillum and Pseudomonas were studied and applied in certain crops systems as bio-fertilizers (Naiman et al. 2009; Piccinin et al. 2011) and to ameliorate salinity stress in numerous economic crops (Egamberdieva 2005; Saghafi et al. 2019).

Flax (*Linum usitatissimum* L.) is a dicotyledonous plant that belongs to the family Linaceae. It is an economically valuable fiber crop and oilseed (linseed) plant grown all over the world, being it an important source of natural fibers and industrial oil, and can meet the needs of edible oil and protein deficiency (Bassuany et al. 2015). The need to grow flax is mainly due to its high content of proteins, essential oils, lignans, and fibers (Sadak and Dawood 2014). Natural fibers from flax are commonly used in the material industry; also have good potential as strengthening agents in compound polymers. Flax crops are grown, especially in Egypt, for the extracted oils from their seeds and fibers from their stems. Over the last 20 years, the cultivated area has decreased from 60,000 acres to 30,000 acres due to strong competition from other economic winter crops resulting in a difference between consumption and production (Hussein 2007; Ibrahim 2009). Therefore, it is necessary to improve the tolerance of flax plants to face the deleterious effects of salinity, which could be achieved by using endophytic bacterial isolates (*A. brasilense* and *P. geniculata*). From this point, our study was conducted to evaluate the possible role of these endophytes in salinity tolerance enhancements of flax plants by measuring some parameters such as growth aspects, photosynthetic pigments, soluble sugars, soluble proteins, free proline, total phenols, ascorbic acid, minerals (Na^+^ and K^+^), the activity of antioxidant enzymes (superoxide dismutase, peroxidase, and ascorbate peroxidase), lipid peroxidation, hydrogen peroxide contents.

## Materials and methods

### Collection, isolation and identification of endophytic bacteria

Two endophytic bacterial isolates were used to face salinity-induced stress in flax plants. Samples were collected, put into plastic bags in ice box for the isolation of potential endophytic bacteria according the described methods by Petrini et al. (1992) and Werner et al. (1997) in which samples were immersed two times in 70% ethanol for three minutes and immersed twice in 2–4% aqueous solution of sodium hypochlorite for five minutes and immersed again for one minute in 70% ethanol. Finally, samples were washed two times in sterile distilled water for five minutes to remove surface sterilization agents with further drying in sterilized paper in a laminar flow hood. Subsequently, sterilized plant samples were crushed under sterile conditions and the resulting juices were plated on King’s agar medium or N-free semisolid malate medium for solation of Pseudomonas and Azospirillum spp., respectively. About 1 ml of the macerated tissue was serially diluted to 10^–3^ using sterile potassium phosphate buffer (10 mM; pH 7). About 1 ml from each dilution of intercellular fluid of its tissue was plated in triplicate and kept in an incubator at 28 °C for 48 h. One of them was *Azospirillum brasilense*, which was isolated from the stem of a cultivated-barley plant in Ras-Sudr, South of Sinai, Egypt. While the other isolate was *Pseudomonas geniculata* strain, which was isolated from the stem of a cultivated-wheat plant in El-Maghra region—North of Sinai, Egypt. Depending on the molecular level, both strains were identified using a partial 16S rRNA gene sequence technique according to Berg et al. (2002) at Sigma Scientific Services Co. (Giza, Egypt). *Pseudomonas geniculata* strain 60786 ATCC 19374, GenBank assigned accession number is NR117678. *Azospirillum brasilense* strain 7000 ATCC 29145 accession number is KU97890. Selective medium used for Azospirillum was N-free semisolid malate medium (NFb medium) (Baldani and Döbereiner 1980). While, the medium used for Pseudomonas was King Agar B (Sigma-Aldrich) (King et al. 1954). The isolated strains were considered as plant growth-promoting bacteria (PGPB) because they exhibited different plant promoting activities as phosphate dissolving activities in terms of phosphatase activity, pH reduction, secretion of multiple organic acids as ascorbic acid, citric acid, formic acid, Lactic acid, malic acid, oxalic acid and succinic acid, indole acetic acid production reaching 106 and 174 ppm for *A. brasilense* and *P. geniculata*, respectively. Only *A. brasilense* exhibited nitrogenase activities reaching 96 n.mole C_2_H_4_/ml/h (Omer 2016, 2017).

### Microbiological analysis of flax rhizosphere

Five plants from each pot were collected and stored at 4 °C until processing in the laboratory. Standard microbiological methods were used to isolate bacteria from the rhizosphere. Samples were serially diluted and plated onto two selective media. Microbiological characteristics of the flax rhizosphere were measured in terms of microbial densities and CO_2_ evolution. Microbial densities as total microbial count and that of *Azospirillum* and *Pseudomonas spp.* were estimated using Nutrient, Doberiner and King media, respectively. CO_2_ evolution (μg/g dry soil/h) in the rhizosphere soil were determined according to Parmer and Schmidt (1964).

### Experiment layout

A pot study was undertaken in the greenhouse of the Desert Research Center's microbiological unit in Cairo, Egypt. Under normal conditions such as temperature, humidity, and light. Flax (*Linum usitatissimum* L. cv. Giza 9) seeds were obtained from Agricultural Research Center (ARC), Giza, Egypt. Microbial inoculants of *A. brasilense*, *P. geniculata*, and co-inoculation of both strains were used for treating flax plants. The present experiment was performed in a completely randomized design (CRD) with four replicates; the treatments and its replicates were randomly distributed within the study area. Ten seeds of flax crops were planted in pots (30 cm in diameter) containing 10 kg of salinized clay soil obtained from Sahl El-Tina, Sinai, Egypt. The characteristic features of the collected soil were as follows: soil depth 0–30 cm^−1^, texture clay, E.C. 11.3 dS/m, pH 7.4, HCO_3_^−^ 17.93 mg g^−1^, Cl^−^ 51.03 mg g^−1^, ${\text{SO}}_{4}^{ -2 }$ 9.23 mg g^−1^, Ca^2+^ 20.53 mg g^−1^, Mg^2+^ 14.53 mg g^−1^, Na^+^ 55.63 mg g^−1^, and K^+^ 0.60 mg g^−1^. Flax seeds were applied to the selected endophytic bacterial inoculum (10^6^ CFU/ml) using carboxymethyl cellulose (CMC) solution (5%) in the range of 500 g seeds/125 ml of inoculum mixed with 25 ml of CMC. Regarding co-inoculation application, both bacterial strains were counted separately, then added to the soil. Uniform seeds were selected to dry in shade before planting (Samasegaran et al. 1982). Control treatment seeds were retained. After seedling growth, the plants were thinned to four plants per pot and then each pot was inoculated with 10 ml of bacterial inoculum (10^6^ CFU/ml) of endophytic bacterial strains (*A. brasilense*, *P. geniculata*) and their co-inoculation. Pots were set as follows: i- Saline soil control, ii- saline soil + *A. brasilense*, iii- saline soil + *P. geniculata*, and iv- saline soil + mixture of *A. brasilense* and *P. geniculata*. After 9 weeks of planting, plant samples were collected for morphological and physiological parameters.

### Morphological characteristics

Lengths (cm) of shoots and roots, fresh weights (g) of shoots and roots, dry weights (g) of shoots and roots, as well as number of leaves of flax plants were manually estimated using a ruler and a scale.

### Photosynthetic pigments determination

The chlorophyll and carotenoids contents of fresh leaves of flax plants were determined using a method described by Vernon and Seely (1966) and Lichtenthaler (1987) respectively. In this method, fresh leaves (1 g) were extracted in 100 ml acetone (80%) followed by filtration. The filtrate was put into an adequate volumetric flask and completed with acetone (80%) to reach 100 ml. The optical density (O.D.) of the filtrate was measured at 665, 649, and 470 nm. Calculate chlorophylls and carotenoids contents as the following equations:

$${\text{Chlorophyll a }} = \, \left[ {\left( {{11}.{63 } \times {\text{ O}}.{\text{D}}.{665}} \right) \, - \, \left( {{2}.{39 } \times {\text{ O}}.{\text{D}}.{649}} \right)} \right].$$

$${\text{Chlorophyll b }} = \, \left[ {\left( {{2}0.{11 } \times {\text{ O}}.{\text{D}}.{649}} \right) \, - \, \left( {{5}.{18 } \times {\text{ O}}.{\text{D}}.{665}} \right)} \right].$$

$${\text{Chlorophylls }}\left( {{\text{a}} + {\text{b}}} \right) \, = \, \left[ {\left( {{6}.{45 } \times {\text{ O}}.{\text{D}}.{665}} \right) \, + \, \left( {{17}.{72 } \times {\text{ O}}.{\text{D}}.{649}} \right)} \right].$$

$${\text{Carotenoids }} = \, \left[ {\left( {{1}000 \, \times {\text{ O}}.{\text{D}}.{ 47}0} \right) \, - \, \left( {{1}.{82 } \times {\text{ Chl}}.{\text{ a}}} \right) \, - \, \left( {{85}.0{2 } \times {\text{ Chl}}.{\text{ b}}} \right)} \right]/{198}{\text{.}}$$

The unit mg/g fresh weight (FW) was used to express about these contents.

### Osmolyte contents determination

A described method of Umbreit et al. (1964) was applied for the determination of sugar contents in the dried tissues of flax plants. After the plant tissue was dried and grinded, one gram of it was homogenized with 5 ml of phenol (2%) and 10 ml of trichloroacetic acid (30%). After filtration, mix 2 ml of filtrate with 4 ml of anthrone reagent (2 g anthrone in 1000 ml of 95% sulfuric acid). The developed blue green color was measured using a spectrophotometer at 620 nm.

To assay, the soluble proteins in the dried tissues of flax plants, the method of Lowry et al. (1951) was carried out. The dried sample (0.1 g) was extracted with 5 ml of diluted phenol (2%) and 10 ml of distilled water. After filtration, 1 ml of the extract mixed with 5 ml of alkaline reagent [50 ml from solution A (50 ml of sodium carbonate (2%) dissolved in 0.1 N sodium hydroxide) in addition to 1 ml from solution B (0.5 g copper sulfate dissolved in 1% sodium potassium tartrate)]. Finally, 0.5 ml of the diluted folin phenol reagent (1:3 v/v) was added. The developed color after 30 min measured at 750 nm.

The procedure of Bates et al. (1973) was followed for the estimation of free proline contents in the dried tissues of flax plants. In such a method, 0.5 g of dried plant material was homogenized in 10 ml of sulfosalicylic acid (3%). After filtration, 2 ml of filtrate reacted with 2 ml acid ninhydrine (1.25 g of ninhydrine in 30 ml of glacial acetic acid and 20 ml of 6 M phosphoric acid, with agitation, until dissolved) and 2 ml of glacial acetic acid. This reaction was placed in a boiling water bath for one hour, then placed in an ice bath. Finally, the reaction mixture extracted with 4 ml of toluene. The chromophore containing toluene was aspirated from the aqueous phase and read spectrophotometrically at 520 nm.

### Estimation of ascorbic acid and total phenol contents

To determine the contents of ascorbic acid in the dry shoots of flax plants, half a gram was ground with liquid nitrogen and suspended in 2 ml of trichloroacetic acid (5%) and then exposed to cool centrifugation 15 min at 10,000 rpm. The supernatant (2 ml) was mixed with 8 ml of trichloroacetic acid (10%). The mixture was shaken and kept in an ice bath for 5 min and centrifuged for another 5 min at 3000 rpm. Finally, 5 ml of the extract was reacted with 2 ml of distilled water and 2 ml of Folin’s reagent. After 10 min, the appeared blue color was measured spectrophotometrically at 760 nm (Jagota and Dani 1982).

The phenolic content value was observed in dried shoots of flax plants using the procedure described by Dai et al. (1993) as the following: 1 g of plant tissue was extracted in 5–10 ml of ethanol (80%) for at least 24 h. After filtration, the residue was re-extracted twice times with the same solvent. All extracts were completed to 50 ml with ethanol (80%). The extract (0.5 ml) was mixed well with 0.5 ml of Folin’s reagent followed by shaking for 3 min. Saturated Na_2_CO_3_ solution (1 ml) then distilled water (3 ml) were added and homogenized well. After one hour, the developed blue color was measured using a spectrophotometer at 725 nm.

### Lipid peroxidation (MDA content) estimation

The described steps in the method of Heath and Packer (1968) were followed for the determination of malondialdehyde contents (as an indication of lipid peroxidation) in fresh leaves of flax plants. Samples were homogenized with trichloroacetic acid (5%) and centrifugated for 10 min at 4000 rpm. After filtration, 2 ml of the extract was reacted with 2 ml of Thiobarbituric acid (0.6%) solution, then the mixture was placed in a water bath for 10 min until cooled. Read the developed color at 532, 600, and, subsequently, 450 nm.

### ***Hydrogen peroxide (H***_***2***_***O***_***2***_***) content determination***

The contents of hydrogen peroxide (H_2_O_2_) in fresh leaves of flax plants were estimated according to the described procedure in Mukherjee and Choudhuri (1983) method. In which, fresh samples (0.05 g) were homogenized with 4 ml cold acetone. Then, 3 ml of the extracted solution were mixed with 1 ml of titanium dioxide (0.1%) in H_2_SO_4_ (20%) (v:v). This mixture was centrifuged for 15 min at 6000 rpm. The developed yellow color of the supernatant was spectrophotometrically measured at 415 nm.

### ***Sodium (Na***^+^***) and potassium (K***^+^***) content determination***

Dry flax shoot samples (0.1 g) had been digested for 12 h with a solution of perchloric acid (80%) and concentrated sulfuric acid (1:5). The described technique of Williams and Twine (1960) was used to measure sodium (Na^+^) and potassium (K^+^) contents of the digested samples using flame photometry.

### Antioxidant enzymes assay

For enzymes extraction from terminal buds of flax plants, methods of Mukherjee and Choudhuri (1983), Amin et al. (2021) and Abdel Latef et al. (2020) were used for extraction of superoxide dismutase (SOD), peroxidase (POD) and ascorbate peroxidase (APX).

Superoxide dismutase (E.C.1.15.1.1) activity was determined by measuring the repression of pyrogallol auto-oxidation as described in Marklund and Marklund (1974). The solution (10 ml) consisted of 3.6 ml of distilled water, 0.1 ml of enzyme, 5.5 ml of 50 mM phosphate buffer (pH 7.8), and 0.8 ml of 3 mM pyrogallol (dissolved in 10 mM HCl). The rate of pyrogallol reduction was measured with a UV spectrophotometer at 325 nm.

Peroxidase (E.C.1.11.1.7) activity was assayed using solution containing 5.8 ml of 50 mM phosphate buffer pH 7.0, 0.2 ml of the enzyme extract and 2 ml of 20 mM H_2_O_2_ after addition of 2 ml of 20 mM pyrogallol, the rate of increase in absorbance as pyrogallol was determined spectrophotometrically by UV–visible spectrophotometer within 60 s at 470 nm and 25 °C (Bergmeyer 1974; Badawy et al. 2021a).

For determination of ascorbate peroxidase (E.C.1.11.1.11) activity, 0.2 ml of enzyme extract mixed with 0.5 mM of ascorbic acid, 0.8 ml of potassium phosphate buffer (50 mM; pH 7), 0.1 mM H_2_O_2_. The changes in absorbance were spectrophotometrically read at 290 nm (Chen and Asada 1992).

### Statistical analysis

Statistical analysis was performed by one-way variance analysis (ANOVA) to assess a relevant difference between different treatments using CoStat (CoHort software, Monterey, CA, USA). The least significant difference (LSD) at P ≤ 0.05 has been used to imply a significant difference among treatments. Principal components analysis (PCA) was conducted using Minitab (version 19.0).

## Results

### Microbiological characteristics of flax plants rhizosphere

Results in Table 1 showed that generally, a remarkable increase in the microbial densities of flax plants rhizosphere was recorded as a result of bacterial inoculation regardless of the type of bacteria used. Total microbial count increases reached 93.4% due to mixed bacterial inoculation. Inoculation with any endophytic bacteria had a positive effect on the counts of both *Azospirillum* spp. and *Pseudomonas* spp. in the rhizosphere region compared to uninoculated ones. The same patterns were detected concerning CO_2_ evolution as an indicator of microbial activity in the rhizosphere, where the maximum increase was measured in plants inoculated with a mixture of bacterial inoculum (138.5%).

**Table 1:** Microbiological characteristics of Flax (*Linum usitatissimum*) rhizosphere at the harvest

Treatment	TBC	Increasing In TBC (%)	Az. C	Increasing In Az. Count (%)	Ps. C	Increasing in Ps. Count (%)	CO_2_	Increasing in CO_2_ (%)
Control	74 ± 5c	23.3	1.7 ± 0.58c	54.5	119 ± 20.6b	32.2	10.6 ± 0.41c	51.4
*A. brasilense*	102 ± 12b	70.1	2.9 ± 0.81a	163.6	120 ± 19.7b	33.3	16.1 ± 0.45b	130
*P. geniculata*	107 ± 22.3b	78.3	2.4 ± 0.52b	118	210 ± 18c	133.4	16.3 ± 0.4b	132.8
Mixture	116 ± 14a	93.4	2.8 ± 0.45a	154.5	204 ± 12a	126.6	16.7 ± 0.45a	138.5

TBC × (10^5^ CFU/g dry soil): Total bacterial count (10^5^ CFU/g dry soil), Az. C. × (10^3^ CFU/g dry soil): *Azospirillum* spp. Count, Ps. C. × (10^3^ CFU/g dry soil): *Pseudomonas* spp. Count and Co_2_ (µg g^−1^ dry soil)

### Growth criteria

**Fig. 1:**
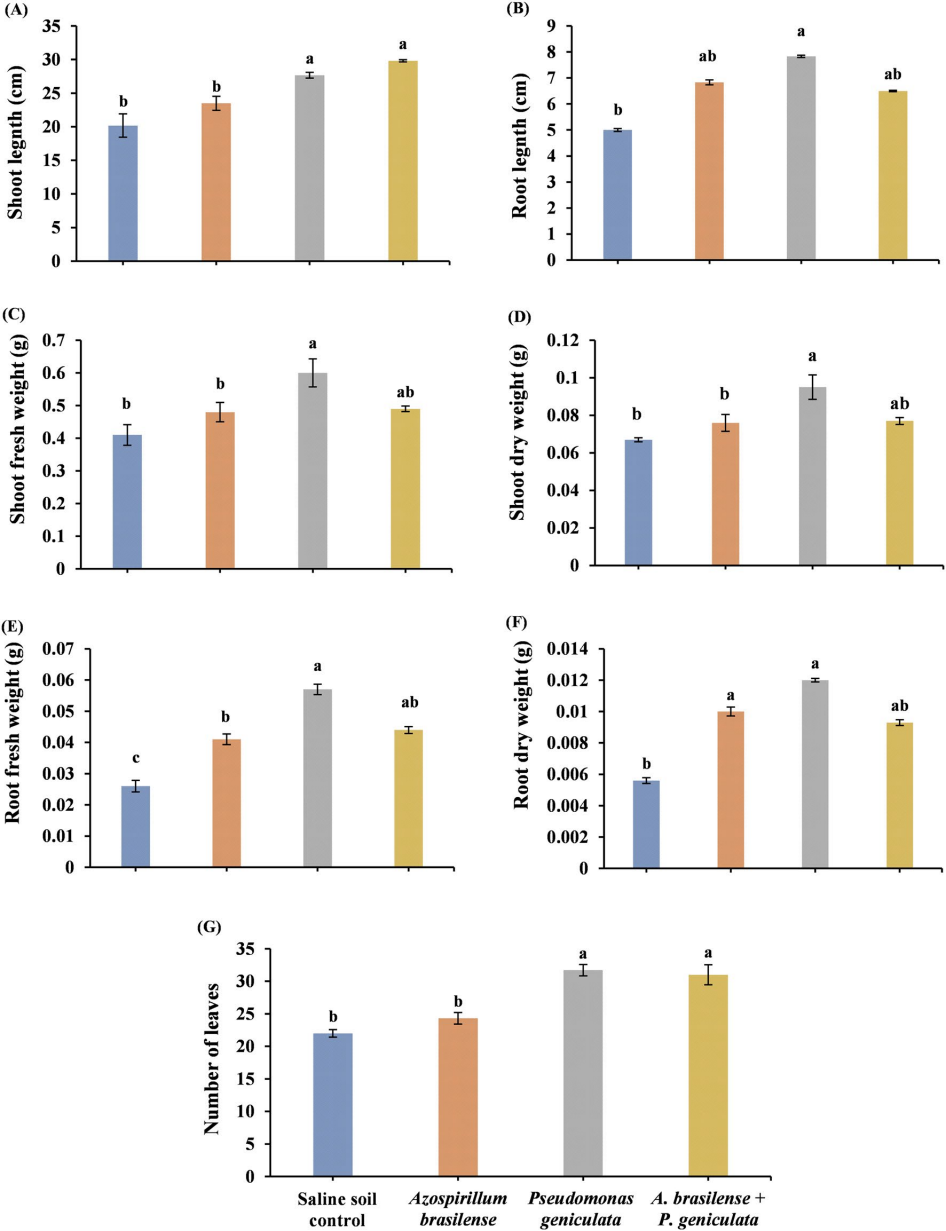
Effect of the inoculation with *Azospirillum brasilense*, *Pseudomonas geniculata* individually or co-inoculation on growth parameters (**A**) shoot length; (**B**) root length; (**C**) shoot fresh weight; (**D**) shoot dry weight; (**E**) root fresh weight; (**F**) root dry weight; and (**G**) number of leaves of flax plants (*Linum usitatissimum*) grown in saline soil. Bars represent the mean of three replicates (n = 3), vertical bars indicate ± standard error. Different letters indicate significant difference among means according to LSD test at p < 0.05

Under salinity stress conditions, inoculation of flax plants with endophytic bacterial isolates (*A. brasilense* and *P. geniculata*) showed different responses in their growth characteristics (Fig. 1). Bacterial strain *A. brasilens*e inoculation enhanced the growth parameters such as shoot length, root length, fresh weight of shoot, dry weight of shoot, fresh weight of root, dry weight of root, and number of leaves by 16.5%, 36.6%, 17.07%, 13.43%, 57.7%, 78.6%, and 10.5% respectively. Additionally, application of the bacterial strain *P. geniculata* significantly increased the aspects of growth such as shoot length, root length, fresh weight of shoot, dry weight of shoot, fresh weight of root, dry weight of root, and number of leaves about 37.2%, 56.6%, 46.3%, 41.8%, 119.2%, 114.3%, and 44.9% respectively when compared with control (plants grown in salinized soil). Furthermore, co-inoculation bacterial species *A. brasilense* and *P. geniculata* markedly enhanced the growth criteria of salinity-stressed flax plants represented in shoot length, root length, fresh weight of shoot, dry weight of shoot, fresh weight of root, dry weight of root, and number of leaves about 47.9%, 30%, 19.5%, 14.9%, 69.2%, 66.1%, and 40.9% respectively.

### Photosynthetic pigments

**Fig. 2:**
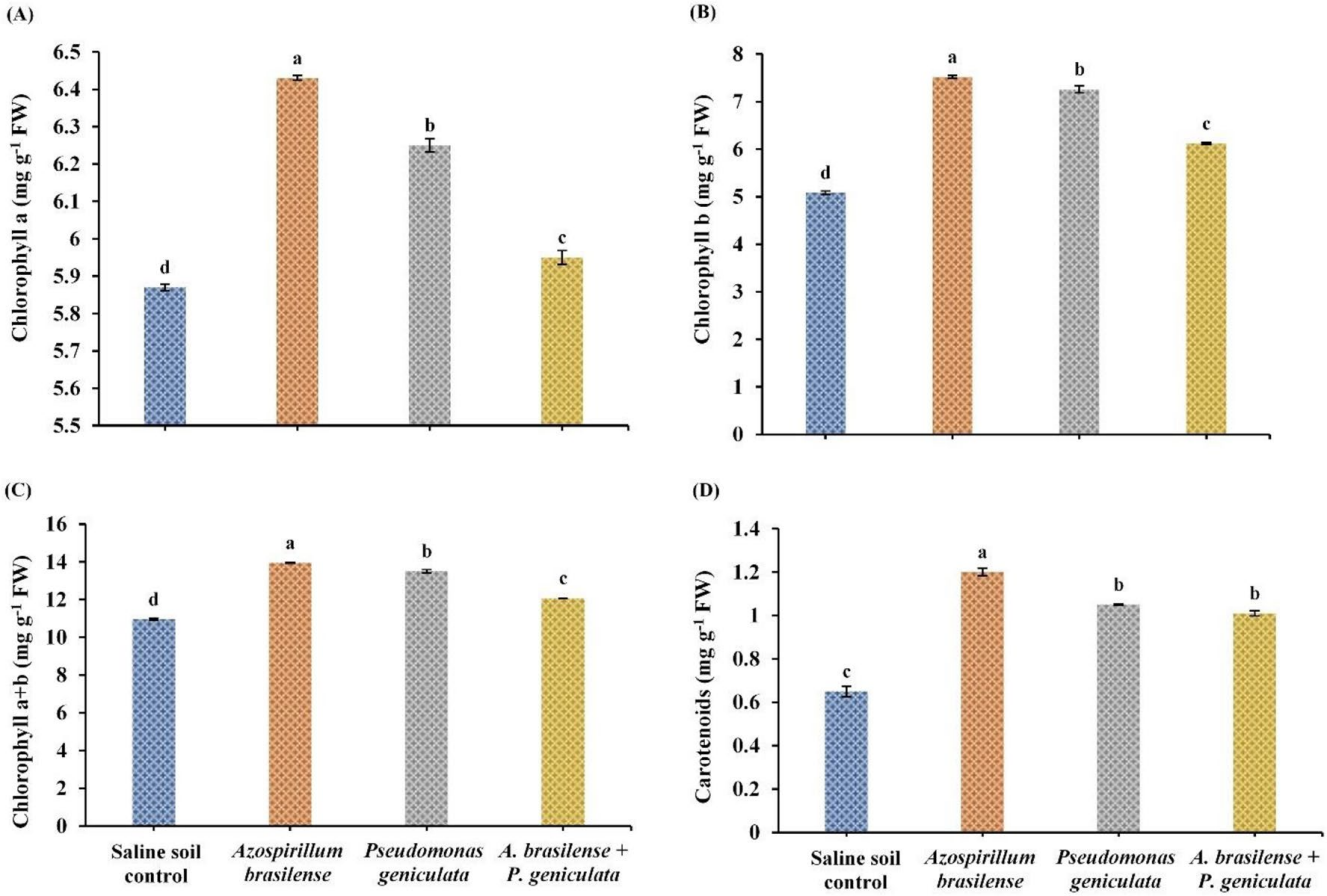
Effect of the inoculation with *Azospirillum brasilense*, *Pseudomonas geniculata* individually or co-inoculation on Photosynthetic pigments (mg g^−1^ FW) (**A**) Chlorophyll a; (**B**) Chlorophyll b; (**C**) Chlorophyll a + b and (**D**) Carotenoids of flax plants (*Linum usitatissimum*) grown in saline soil. Bars represent the mean of three replicates (n = 3), vertical bars indicate ± standard error. Different letters indicate significant difference among means according to LSD test at p < 0.05. *FW* fresh weight

The observed results in Fig. 2 clarified that chlorophylls and carotenoids were varied in their contents in salinity-stressed flax plants due to the inoculation with the endophytic bacterial strains *A. brasilense* and *P. geniculata* (individually or in co-inoculation). Flax plants that were grown under salinity stress conditions and inoculated with the bacterial isolate *A. brasilense* observed significant increases in photosynthetic pigments, e.g., Chl. *a*, Chl. *b*, Chl. *a* + *b* and carotenoids by 9.5%, 48%, 24.4%, 84.6% respectively, when compared to un-inoculated and salinized control plants. Moreover, treating stressed flax plants with endophytic bacterial strain *P. geniculata* displayed significant enhancements in Chl. *a*, Chl. *b*, Chl. *a* + *b* and carotenoids by 6.47%, 42.9%, 23.29% and 61.54%, respectively. Dual inoculation with the two endophytic bacteria (*A. brasilense* and *P. geniculata*) significantly increased the contents of Chl. *a* (1.36%), Chl. *b* (20.47%), Chl. *a* + *b* (10.23%) and carotenoids (55.38%).

### Osmolytes

**Fig. 3:**
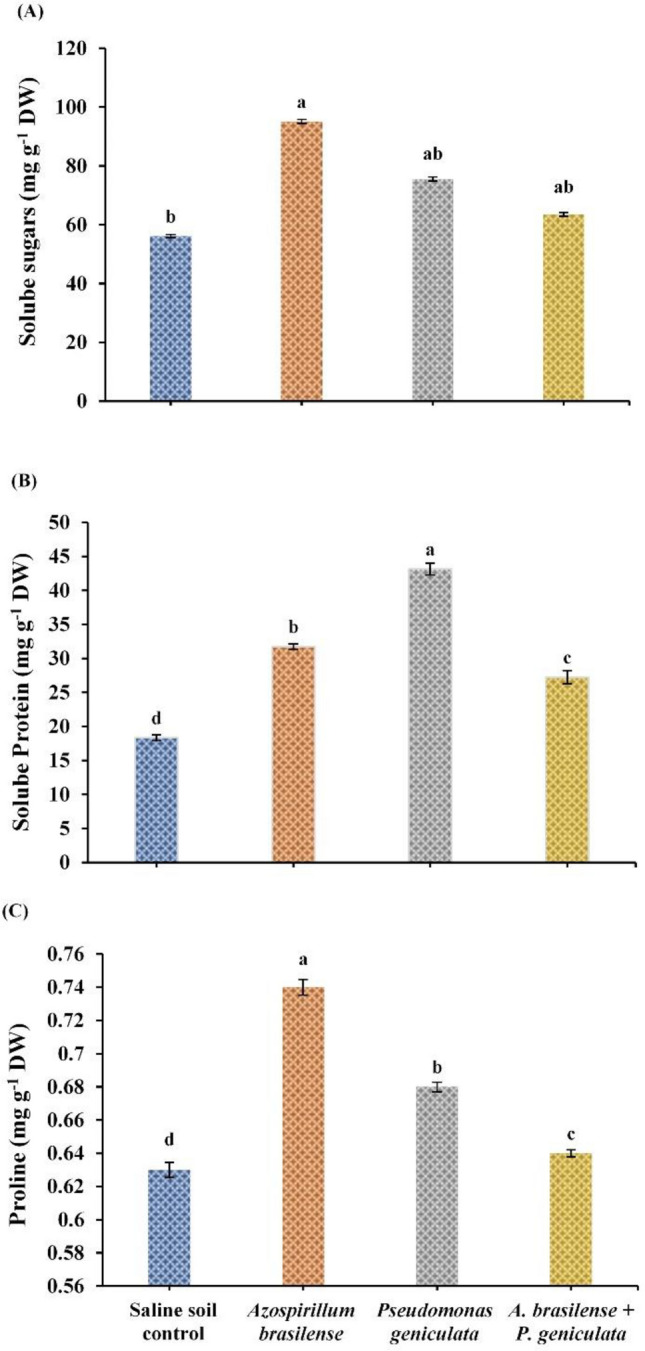
Effect of the inoculation with *Azospirillum brasilense*, *Pseudomonas geniculata* individually or co-inoculation on Soluble sugars; Soluble protein and proline contents of flax plants (*Linum usitatissimum*) grown in saline soil. Bars represent the mean of three replicates (n = 3), vertical bars indicate ± standard error. Different letters indicate significant difference among means according to LSD test at p < 0.05. *DW* dry weight

The response of salinity-stressed flax plants to inoculation with endophytic bacterial isolates was displayed in Fig. 3. Significant increases were recorded in the contents of soluble sugars, soluble proteins, and free proline in stressed-flax plants as a result of inoculation with the endophyte *A. brasilense* by 69.5%, 73.1%, and 17.46%, respectively. *Pseudomonas geniculata* inoculation insignificantly increased sugar contents about 34.6% and significantly increased protein and proline contents by 135.35% and 7.94%, respectively, in salinity-stressed flax plants when compared with the un-inoculated plants. Co-inoculation with two bacterial strains (*A. brasilense* and *P. geniculata*) insignificantly increased the contents of sugars by 13.19% while significantly increased the contents of soluble protein and free proline by 48.60% and 1.58% respectively, in shoots of stressed-flax plants.

### Total phenols and ascorbic acid

**Fig. 4:**
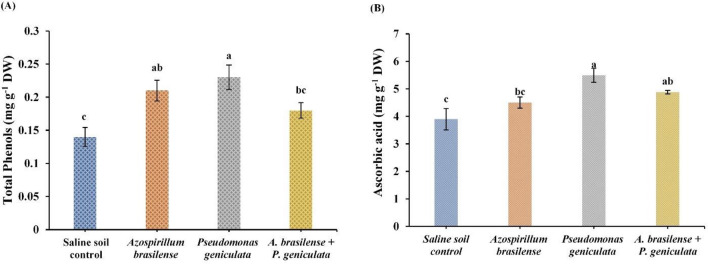
Effect of the inoculation with *Azospirillum brasilense*, *Pseudomonas geniculata* individually or co-inoculation on (**A**) Total Phenols (mg g^−1^ DW); (**B**) Ascorbic acid (mg g^−1^ DW) contents of flax plants (*Linum usitatissimum*) grown in saline soil. Bars represent the mean of three replicates (n = 3), vertical bars indicate ± standard error. Different letters indicate significant difference among means according to LSD test at p < 0.05. *FW* fresh weight

The impact of endophytic bacterial isolates (*A. brasilense* and *P. geniculata*) inoculation on total phenols and ascorbic acid content in flax plants grown in salinized soil was demonstrated in Fig. 4. Application of endophytic bacteria *A. brasilense* significantly increased the contents of total phenols by 50% and ascorbic acid by 15.38% in flax plants grown under salinity stress conditions. Moreover, salinity-stressed flax plants that were inoculated with the bacterial strain *P. geniculata* observed a significant increase in the contents of total phenols by 64.28% and ascorbic acid by 40.67% when compared to un-inoculated stressed plants. Co-inoculation with two bacterial strains *A. brasilense* and *P. geniculata* diminished salinity stress effects in flax plants by raising total phenols and ascorbic acid contents by 28.57% and 25.12% respectively.

### ***MDA and H***_***2***_***O***_***2***_

**Fig. 5:**
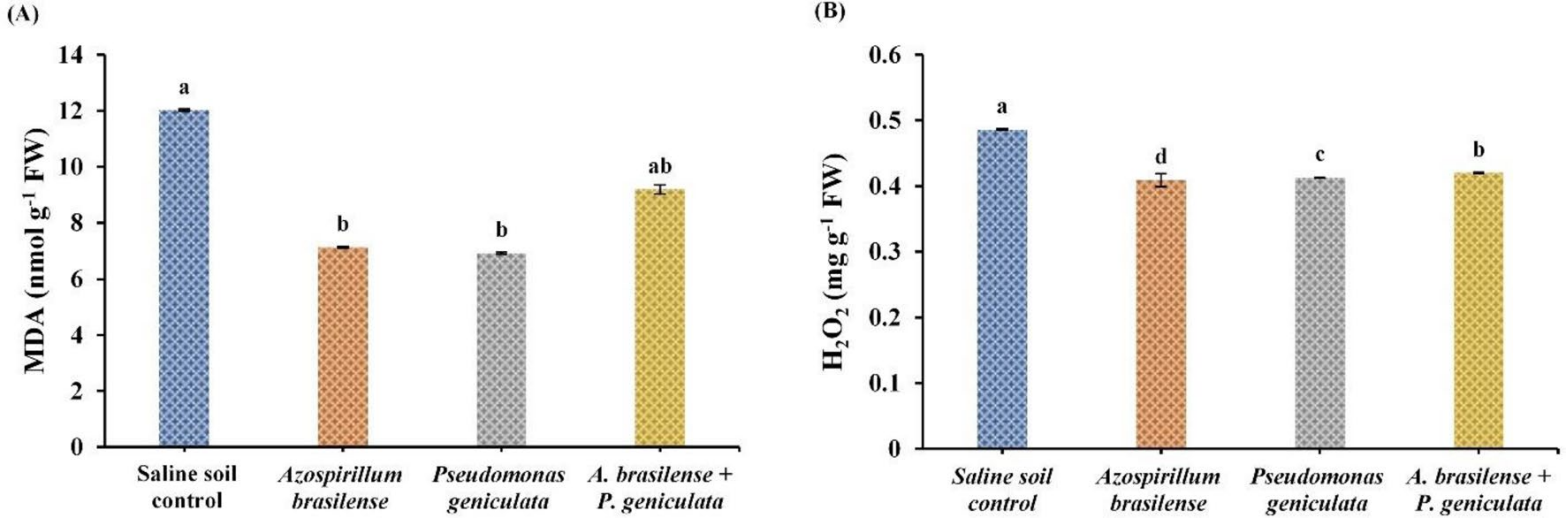
Effect of the inoculation with *Azospirillum brasilense*, *Pseudomonas geniculata* individually or co-inoculation on (**A**) MDA (nmol g^−1^ FW); (**B**) H_2_O_2_ (mg g^−1^ FW) contents of flax plants (*Linum usitatissimum*) grown in saline soil. Bars represent the mean of three replicates (n = 3), vertical bars indicate ± standard error. Different letters indicate significant difference among means according to LSD test at p < 0.05. *FW* fresh weight

Effect of endophytic bacterial isolates (*A. brasilense* and *P. geniculata*) inoculation on the contents of malondialdehyde and hydrogen peroxide in flax plants grown in salinized soil was shown in Fig. 5. Application of endophytic bacteria *A. brasilense* significantly decreased the contents of MDA by 40.77% and H_2_O_2_ by 15.85% in flax plants grown under salinity stress conditions. Moreover, salinity-stressed flax plants that were inoculated with the bacterial strain *P. geniculata* observed significant decreases in the contents of MDA by 42.52% and H_2_O_2_ by 15.03% when compared to un-inoculated stressed plants. Dual inoculation with two bacterial strains *A. brasilense* and *P. geniculata* diminished salinity stress effects in flax plants by declining MDA and H_2_O_2_ contents by 23.55% and 13.59%, respectively.

### Minerals

Under salt stress, flax plants inoculated with *A. brasilense* and *P. geniculata*, as well as their mixture, significantly reduced sodium (Na^+^) content by 42.74%, 52.75%, and 46.53%, respectively (Table 2). However, potassium (K^+^) content was significantly increased by 36.67%, 63%, and 43.57, respectively (Table 2), in response to the above-mentioned treatments when compared to saline soil control plants.

**Table 2:** Effect of the inoculation with *Azospirillum brasilense*, *Pseudomonas geniculata* individually or co-inoculation on sodium (Na^+^) and potassium (K^+^) content (mg g^−1^ DW) of flax plants (*Linum usitatissimum*) grown in saline soil

Treatment	Na^+^	K^+^
Saline soil control	17.69 ± 0.16a	3.19 ± 0.02d
*A. brasilense*	10.13 ± 0.014b	4.36 ± 0.04c
*P. geniculata*	8.36 ± 0.13d	5.20 ± 0.02a
Mixture	9.46 ± 0.02c	4.58 ± 0.04b

Bars show means of three independent replications (n = 3) ± standard error. Different letters indicate significant difference among means according to LSD test at p < 0.05

*DW* dry weight

### Antioxidant enzymes

Activities of antioxidant enzymes in salinity-stressed flax plants that were inoculated with the endophytes *A. brasilense* and *P. geniculata* were clarified in Table 3. Treating flax plants with *A. brasilense* inoculum enhanced the activities of antioxidant enzymes SOD, POD, and APX by 50%, 71.29%, and 41.91% respectively, when compared with control plants (grown in salinized soil without bacterial inoculation). Also, endophytic bacterial strain *P. geniculata* significantly increased antioxidant enzymes activities in flax plants such as SOD, POD, and APX by 125%, 77.77% and 49.26%, respectively. Co-inoculation with the two bacterial strains *A. brasilense* and *P. geniculata* showed insignificant enhancements in the activity of SOD by 8.33%, POD by 21.29% and APX by 18.38%.

**Table 3:** Effect of the inoculation with *Azospirillum brasilense*, *Pseudomonas geniculata* individually or co-inoculation on Antioxidant enzymes activity (U g^−1^ FW) (**A**) Superoxide dismutase (SOD); (**B**) Peroxidase (POD) and (**C**) Ascorbate-peroxidase of flax plants (*Linum usitatissimum*) grown in saline soil

Treatment	SOD	POD	APX
Saline soil control	6 ± 0.50b	5.4 ± 0.83b	6.8 ± 0.67b
*A. brasilense*	9 ± 1.00ab	9.25 ± 0.80a	9.65 ± 0.55a
*P. geniculata*	13.5 ± 1.00a	9.6 ± 0.15a	10.15 ± 0.30a
Mixture	6.5 ± 0.76b	6.55 ± 0.35b	8.05 ± 0.13ab

Bars show means of three independent replications (n = 3) ± standard error. Different letters indicate significant difference among means according to LSD test at p < 0.05. *FW* fresh weight

### PCA Analysis demonstrated positive and negative relationships among treatments and variables

**Fig. 6:**
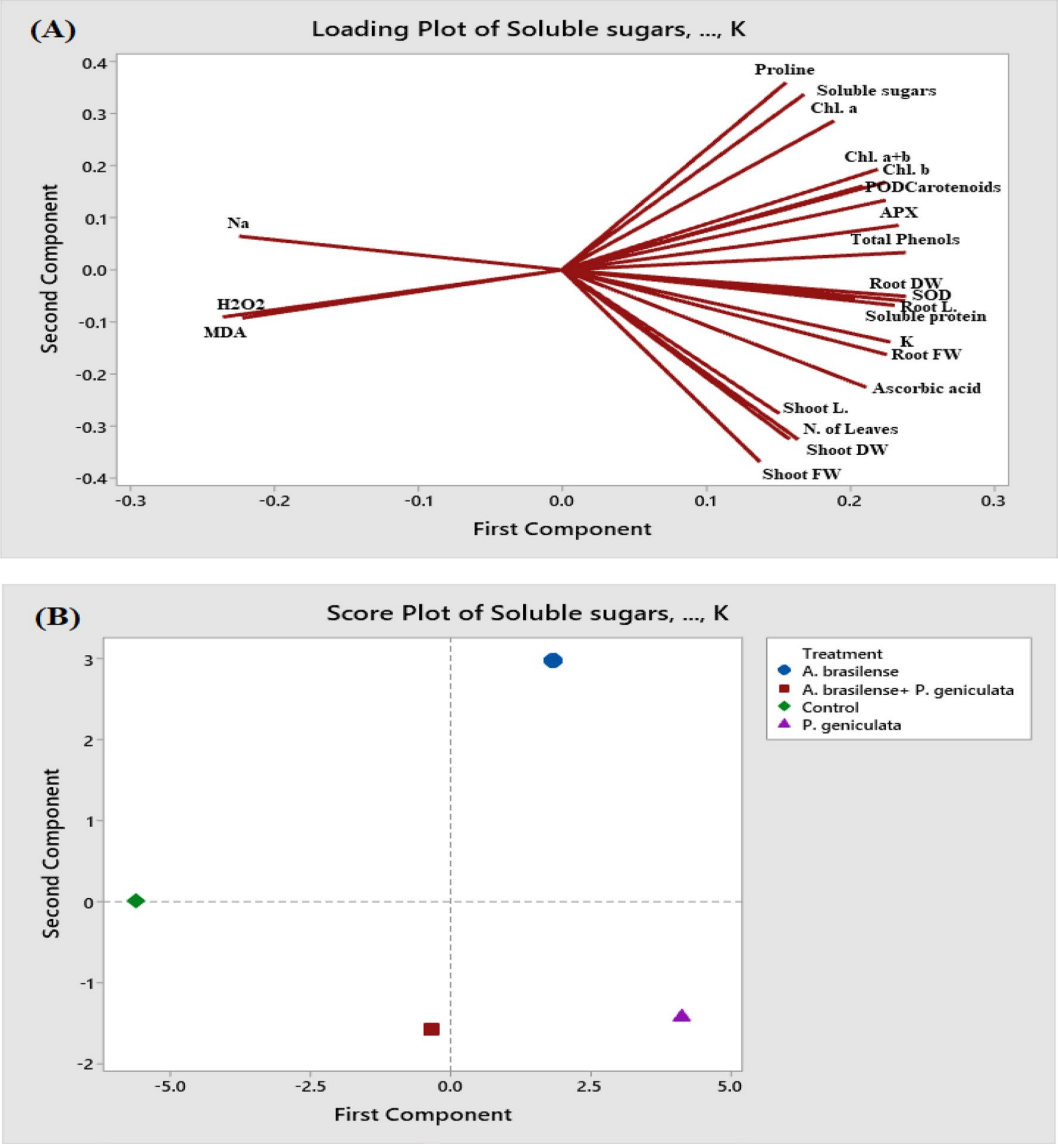
**A** Principal component analysis (PCA) to understand the variability relationships of parameters and treatments in flax plants. The entire dataset was analyzed using a PCA clustering approach. The parameters included Shoot L. (Shoot length), Root L. (Root length), Shoot FW (fresh weight of shoot), Shoot DW (Shoot dry weight), Root FW (fresh weight of root), Root DW (Root dry weight), number of leaves, Chl. *a* (chlorophyll *a*), Chl. *b* (chlorophyll *b*), (carotenoids), soluble sugars, soluble protein, proline, K (potassium), Na (sodium) MDA (malondialdehyde), H_2_O_2_ (Hydrogen peroxide), SOD (superoxide dismutase), APX (ascorbate peroxidase), and POD (peroxidase). (**B**) The treatments of the flax plants were shown in different shapes with different colors under salinity stress conditions. The 1st was flax plants grown in saline soil and represented as green rhombus (Saline soil control). The 2nd was flax plants inoculated with both bacterial strains *Azospirillum brasilense* and *Pseudomonas geniculata* and represented as a red square. The 3rd was flax plants inoculated with *Azospirillum brasilense* and represented as a blue circle. The 4th was plants inoculated with *Pseudomonas geniculata* and represented as a purple triangle

Principal component analysis (PCA) was performed to determine the maximum amount of data variability and to study the interaction between variables and endophytic bacterial strains application. treatments. The two principal components PC1 (73.50%) and PC2 (20.30%) explained a total of 93.80% overall data variability. Loading plots of various variables (Fig. 6A) indicated growth aspects, photosynthetic pigments, osmolytes, total phenols, ascorbic acid and antioxidant enzymes were positively correlated with each other and negatively with MDA, H_2_O_2_ and sodium (Na^+^) content. The score plot (Fig. 6B) indicates *A. brasilense* and *P. geniculata* and their mixture treatments. *P. geniculata* appeared to be the best treatment followed by *A. brasilense* and finally their Co-inoculation.

## Discussion

Salinity tolerance is among the most major challenges facing many countries, especially Egypt. Therefore, it is necessary to find solutions to increase the tolerance of plants in strategies to survive with salinity stress and to be able to grow in salinized areas (Abdel Latef et al. 2020, 2021; Osman and Badawy 2020). Recently, the spotlight has been on the use of bio-stimulants and microorganisms to face salt stress and alleviate its deleterious effects on the life of the plant (Abdel Latef et al. 2020; Osman et al. 2021). Endophytic bacteria have been identified as a group of beneficial free-living soil bacteria that symbiotically live inside plant tissues (Ahemad and Kibret 2014). The common traits of endophytic bacteria for plant promotion are the production of plant growth regulators (as auxin, gibberellin, and ethylene), siderophores, induced systemic tolerance, and biocontrol potential (Omer 2016, 2017). Inoculation of wheat plants with salt-tolerant *Azospirillum lipoferum* isolates decreased the harmful effect of salinized soil stress. As the inoculation enhanced the dry weights, number and dry weight of branches, total chlorophyll, sodium, and potassium content of wheat plants (El-Akhdar et al. 2019). The endophytic bacterial strain of *P*. *geniculata* had beneficial effects on the plant growth of various seeds. It also affected the malondialdehyde content and the activity of superoxide dismutase and peroxidase (Liu et al. 2019b).

The present study showed that inoculation with endophytic bacteria *A. brasilense*, *P. geniculata*, and their co-inoculation significantly promoted flax growth characteristics (lengths, fresh and dry weights of shoots and roots as well as number of leaves) as a means of mitigating the negative effects of salinity stress. Our findings are consistent with those of Mazhar et al. (2016) and Khalid et al. (2017). The magnitude increased in growth parameters by endophytic bacteria refers to the ability of *A. brasilense* and *P. geniculata* to produce IAA which stimulates flax cell elongation (Ahmed and Hasnain 2014; Abdel Latef et al. 2020). Under different environmental stresses, plant growth-promoting bacteria with the aid of root growth and development, and in particular hair roots, contribute to increased plant water and nutrient uptake (Kaymak 2010) and also morphological characteristics and metabolism by generating a range of hormone substances such as auxin, gibberellin and cytokinin, resulting in increasing water and nutrient uptake (Cassán et al. 2014). Recently, Abdel Latef et al. (2021) in their study recorded significant increases in the growth parameters of salinity-stressed canola plants in response to the inoculation with the bacterial isolates *Azotobacter chroococcum* and/or *Alcaligenes faecalis*.

Results of the mean comparisons recorded increases in all photosynthetic pigments of flax plants under salinity stress conditions. It is also conceivable that an increase in the chlorophyll content of endophytic bacteria treated plants could be due to the development of a variety of microbial metabolites, such as auxin, gibberellin and cytokinin, arising throughout the absorption of water and minerals (Cassán et al. 2014) which help in facilitate photosynthetic materials in plant cells. Bashan et al. (2006) and Enebe and Babalola (2018) found that wheat plants cultivated under salt stress and inoculated with *Azospirillum Brasilense* resulted in increased development of photosynthetic pigments, which would, in turn, led to a significant increase in photosynthetic rates and CO_2_ fixation. Also, Omar et al. (2009) and Bhat et al. (2020) mentioned that application of endophytic bacterial strains under salt stress enhanced the production of auxiliary photoprotective pigments such as zeaxanthin, and β-carotene which may protect Chlorophyll *a* and Chlorophyll *b* from oxidation during exposure to salt stress.

A common influence of salt stress is the production of osmotic stress due to low osmotic potential of saline solution, which limits crop access to soil water (Munns and Tester 2008; Roy et al. 2019). The osmotic stress-induced shortage of water may lead to the loss of turgidity and cell dehydration as well as, eventually, to the death of plants. Osmotic improvement is a key basis for crop adoption to proceed (Farooq et al. 2015). Our results showed that endophytic bacterial isolates *A. brasilense* and *P. geniculata* and their co-inoculation enabled flax plants to maintain considerable levels of total soluble sugars and soluble protein when they were grown under salinity stress conditions. These results implied that *A. brasilense* and *P. geniculata* and their co-inoculation assisted elevation of sugar, proteins, and free proline levels which might have contributed to osmo-tolerance of salt-exposed flax plants by improving water status, stabilizing membranes and protecting enzymes from denaturation (Singh et al. 2015). Moreover, these increases in the contents of soluble sugars and soluble proteins can also act as energy resources for plants under abiotic stress (Rai 2002; Sami et al. 2016).

Proline is among the most prevalent amino acids reported to accumulate regularly in plant cells in relation to salinity stress (Mansour and Ali 2017). In this study, *A. brasilense* and *P. geniculata* and their co-inoculation increased proline contents in the flax plants grown in saline soil. These findings are consistent with the results of (Huang et al. 2013; Gharsallah et al. 2016; Abdel Latef et al. 2018, 2019; Badawy et al. 2021b). Moslemi et al. (2011) increased proline content and increased plant resistance to dehydration have been reported due to inoculation with microbial strains *Azospirillum lipoferum* and *Pseudomonas putida* bacteria. Moreover, those bacterial strains were challenged with reactive oxygen species by modifying the plant's defensive reaction and, as a result, co-operating plants in rising proline content. Mostly on the positive side, proline accumulation has been shown to enhance salt stress tolerance through leading towards osmotic salt stress adjustment in chickpea (*Cicer arietinum*) and canola (*Brassica napus*) (Ahmad et al. 2016; Li et al. 2017; Abdel Latef et al. 2021).

Plant secondary metabolite accumulation serves as an adaptive response in plants to salinity stress. Phenolic compounds and ascorbic acid can scavenge free radicals generated by reactive oxygen species (ROS) which support plants against salinity stress throughout enhancing the antioxidant defense system (Osman et al. 2021; Attia et al. 2021a, b). Furthermore, phenolic compounds can stabilize cell membranes by reducing membrane fluidity, which also leads to much less transportation of free radicals all over membranes, reducing membrane peroxidation (Abdel Latef et al. 2020; Hussein et al. 2022). Endophytic bacterial strains enhanced contents of total phenols and ascorbic acid over salinity-stressed plant. The previously mentioned increases in total phenols and ascorbic acid content are related to decreases in MDA and H_2_O_2_ content. These results are in line with other researchers (Abdel Latef et al. 2021; Ali et al. 2021) who documented the role of plant growth-promoting rhizobacteria in abolishing the deleterious effect of salinity stress throughout increasing phenolic compounds and ascorbic acid contents.

Malondialdehyde content represents as a final metabolite of the lipid peroxidation process, which results in disruption to cell membrane components, especially lipid materials (Attia et al. 2021a). Flax plants inoculated with both endophytic bacterial strains *A. brasilense* and *P. geniculata* and their co-inoculation showed a decline in the contents of malondialdehyde and hydrogen peroxide. These results are in line with other researchers who have documented the role of endophytic bacteria in diminishing oxidative damage caused by salinity stress throughout decreasing MDA and H_2_O_2_ contents (Bharti et al. 2016; Samaddar et al. 2019; Abdel Latef et al. 2021).

The beneficial role of endophytes in eliminating the detrimental effect of salinity stress was presented in the study by limiting the uptake of sodium (Na^+^) and increasing the uptake of potassium content (K^+^). This beneficial role may be related to the exploration that endophytes facilitates the corporation of crucial elements in the soil to be rapidly consumed by the plant (Kang et al. 2014; Bhat et al. 2020) or even to the increasing availability of minerals in the soil rhizosphere as a result of root exudates substances (Abdel Latef et al. 2021).

Antioxidants involve numbers of antioxidant enzymes that play an effective function in protecting plants from oxidative stress damage (Hahm et al. 2017). Enhancing the activities of antioxidant enzymes has supported plants to face and cope with different stresses such as salinity (Ahammed et al. 2019; Tahjib-Ul-Arif et al. 2019; Osman et al. 2021; Dawood et al. 2021). The findings of Abdel Latef et al. (2020) support our study, the authors found that inoculation of salinity-stressed maize plants with *Azospirillum lipoferum* led to enhancements in antioxidant enzyme activities as peroxidases (about 25%) and ascorbate peroxidases (about 41%). In another study, the activity of ascorbate peroxidase was increased in stressed basil (*Ocimum basilicum* L.) plants in response to the inoculation with endophytic bacterial species *Pseudomonas sp*. and *A. brasilense* either individually or in combination (Heidari and Golpayegani 2012). Our results are in agreement with the results of Liu et al. (2019a; b), they stated that activities of SOD and POD were induced in alfalfa plants grown under saline-alkali conditions about 1.20 and 1.14-fold respectively, more than that in control (untreated plants) as a result of inoculation with *Pseudomonas aeruginosa*. It is well known that antioxidant enzymes possess the ability to scavenge the resulted free radicals in the cell during exposure to different abiotic stresses.

To validate our results on the evaluation of bacterial strains in flax plants, the entire data were subjected to a PCA-based clustering method (Fig. 6A, B). Figure 6A indicates the effect of *A. brasilense* and *P. geniculata* on different measured parameters. The growth aspects, photosynthetic pigments, antioxidant enzymes, osmolytes, total phenols, ascorbic acid and potassium (K^+^) were highly and positively linked with two another and negatively with MDA, H_2_O_2_ and sodium (Na^+^) content. Score plots grouped showed the best bacterial effect on flax plants. This figure suggested that plant growth-promoting rhizobacterial strain *P. geniculata* was the best treatment in modulating all parameter studies as this was found in a right rectangle of the first component, whereas the Co-inoculation of *A. brasilense* and *P. geniculata* were found on the left-hand side of the first component.

## Conclusion

From the observed results in the present study, it can be concluded that the tested endophytic bacteria (*Azospirillum brasilense* and *Pseudomonas geniculata*) play an important role in coping with salinity-induced stress throughout promoting morphological parameters, chlorophylls and carotenoids pigments, soluble sugars, proteins, free proline, total phenols, ascorbic acid, and potassium contents. Application of endophytes reduced the oxidative stress by enhancing the activities of antioxidant enzymes (SOD, POD, and APX) in addition to minimizing the contents of malondialdehyde, hydrogen peroxide, and sodium. So, utilization of *Azospirillum brasilense* and *Pseudomonas geniculata* can be recommended as a promising method in salinity stress alleviation in plants, especially the isolate *Pseudomonas geniculata* which observed significant improvements in most parameters.
